# UPLC-ESI-MRM/MS for Absolute Quantification and MS/MS Structural Elucidation of Six Specialized Pyranonaphthoquinone Metabolites From *Ventilago harmandiana*

**DOI:** 10.3389/fpls.2020.602993

**Published:** 2021-01-11

**Authors:** Suphitcha Limjiasahapong, Khwanta Kaewnarin, Narumol Jariyasopit, Sakchai Hongthong, Narong Nuntasaen, Jonathan L. Robinson, Intawat Nookaew, Yongyut Sirivatanauksorn, Chutima Kuhakarn, Vichai Reutrakul, Sakda Khoomrung

**Affiliations:** ^1^Metabolomics and Systems Biology, Department of Biochemistry, Faculty of Medicine Siriraj Hospital, Mahidol University, Bangkok, Thailand; ^2^Siriraj Metabolomics and Phenomics Center, Faculty of Medicine Siriraj Hospital, Mahidol University, Bangkok, Thailand; ^3^Center of Excellence for Innovation in Chemistry (PERCH-CIC), Faculty of Science, Mahidol University, Bangkok, Thailand; ^4^Division of Chemistry, Faculty of Science and Technology, Rajabhat Rajanagarindra University, Chachoengsao, Thailand; ^5^The Forest Herbarium National Park, Wildlife and Plant Conservation Department, Ministry of Natural Resources and Environment, Bangkok, Thailand; ^6^National Bioinformatics Infrastructure Sweden, Science for Life Laboratory, Department of Biology and Biological Engineering, Chalmers University of Technology, Gothenburg, Sweden; ^7^Department of Biomedical Informatics, College of Medicine, University of Arkansas for Medical Sciences, Little Rock, AR, United States

**Keywords:** mass spectrometry, quantitative metabolomics, structural elucidation, natural product, targeted metabolite profiling

## Abstract

Pyranonaphthoquinones (PNQs) are important structural scaffolds found in numerous natural products. Research interest in these specialized metabolites lies in their natural occurrence and therapeutic activities. Nonetheless, research progress has thus far been hindered by the lack of analytical standards and analytical methods for both qualitative and quantitative analysis. We report here that various parts of *Ventilago harmandiana* are rich sources of PNQs. We developed an ultraperformance liquid chromatography–electrospray ionization multiple reaction monitoring/mass spectrometry method to quantitatively determine six PNQs from leaves, root, bark, wood, and heartwood. The addition of standards in combination with a stable isotope of salicylic acid-D_6_ was used to overcome the matrix effect with average recovery of 82% ± 1% (*n* = 15). The highest concentration of the total PNQs was found in the root (11,902 μg/g dry weight), whereas the lowest concentration was found in the leaves (28 μg/g dry weight). Except for the root, PNQ-332 was found to be the major compound in all parts of *V. harmandiana*, accounting for ∼48% of the total PNQs quantified in this study. However, PNQ-318A was the most abundant PNQ in the root sample, accounting for 27% of the total PNQs. Finally, we provide novel MS/MS spectra of the PNQs at different collision induction energies: 10, 20, and 40 eV (POS and NEG). For structural elucidation purposes, we propose complete MS/MS fragmentation pathways of PNQs using MS/MS spectra at collision energies of 20 and 40 eV. The MS/MS spectra along with our discussion on structural elucidation of these PNQs should be very useful to the natural products community to further exploring PNQs in *V. harmandiana* and various other sources.

## Introduction

**FIGURE 1:**
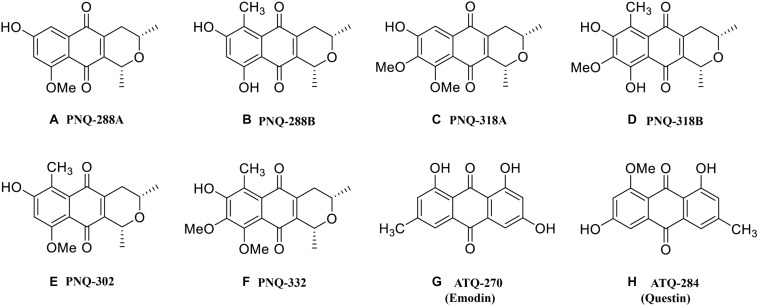
Chemical structures of PNQ and ATQ metabolites from *V. harmandiana* quantified in this study.

Since prehistoric times, humans have continued to use natural products from living organisms including plants, microorganisms, and terrestrial and aquatic biotopes to serve as a rich source of therapeutic products (Brimble et al., 1999; Fabri et al., 2012; Campos et al., 2015; Jiang et al., 2015; Maarisit et al., 2017; Tanahashi et al., 2017; Lemos et al., 2018). Among these, plants provide a massive and unique source of specialized metabolites with a great potential for various pharmaceutical applications (Fang et al., 2019). Specialized metabolites, historically referred to as secondary metabolites, are chemical compounds that are not essential for supporting life of a given organism and are often extracted from medicinal plants for the study of their chemical structures and their biological activities (Khoomrung et al., 2017). The plant *Ventilago* genus of the Rhamnaceae family contains more than 40 species and is found across India, South Asia (Cooke and Johnson, 1963), and recently in Thailand (Kongsriprapan et al., 2012). Various members of the *Ventilago* genus have been used as traditional medicines for numerous therapeutic treatments, e.g., diabetes, wounds, psoriasis, AIDS, diuretics, and arthritis (Kongsriprapan et al., 2012; Molee et al., 2018), as well as anti-inflammation in both animal and cell line studies (Wang et al., 2016; Panthong et al., 2020). *Ventilago harmandiana* Pierre (*V. harmandiana*) is an endemic plant that is found locally in the southern part of Thailand. In over 15 years of our research on the specialized metabolites from this plant, we have discovered 10 novel pyranonaphthoquinones (PNQs) (Supplementary Figures S1A–J), an anthraquinone (ATQ) (ventilanone K, Supplementary Figure S1K), and 10 known anthraquinone (ATQ) derivatives (Supplementary Figures S2A–J) in *V. harmandiana* and *V. maingayi* (Leewanich, 2005; Panthong et al., 2020). Among these, PNQs represent an interesting class of specialized metabolites with a basic skeleton consisting of a fused three-ring structure composed of a pyran, a quinone, and a benzene ring. Previous studies have reported the potential use of PNQs as antibiotics against gram-positive bacteria, fungi, and mycoplasmas (Brimble et al., 1999; Jacobs et al., 2011; Naysmith et al., 2017). Our recent study highlighted the cytotoxic and anti-inflammatory properties of these PNQs isolated from *V. harmandiana.* Specifically, PNQ-318A (Figure 1) demonstrated very promising activity for the future development of anti-inflammatory agents (Panthong et al., 2020).

In practice, the classical approach of identifying PNQs from *V. harmandiana* begins with extraction and fractionation before evaluation of biological activities. Fractions that exhibit promising activity characteristics are further isolated and purified. The purified compounds are subjected to a combination of various characterization techniques, such as nuclear magnetic resonance (NMR), mass spectrometry (MS), and chiroptical spectroscopic methods for structural determination (Wolfender et al., 2019; Panthong et al., 2020). Although these long processes are very effective, they are often complex, tedious, costly, and time consuming. The introduction of metabolomics to research in natural products has brought complementary approaches to the conventional methodology. Recently, metabolite profiling of crude extract by MS and NMR followed by database searching has been suggested to accelerate the discovery and characterization of specialized metabolites (Wang et al., 2016; Wolfender et al., 2019). However, the majority of the studies concentrated primarily on metabolite profiling, metabolite annotation, and database development. Quantitative metabolomics applications (at an absolute level) (Djukovic et al., 2020), on the other hand, are relatively limited. The concentration details of metabolites are a crucial complement to relative quantification (i.e., fold change), which is commonly pursued in natural product and basic metabolomics research. However, the absolute quantification of specialized metabolites in plants can be very challenging because of large chemical diversity, ranging from 5,000 to 10,000 chemical species (Fang et al., 2019), and a wide range of concentrations spanning up to many orders of magnitude (Bueno and Lopes, 2020). Furthermore, the lack of studies using quantitative methods dealing with matrix effects in plant samples hinders further research in this area. Such complex matrices combined with the lack of authentic standards considerably increase the difficulty in achieving high precision and accuracy in quantitative analysis. To date, biochemical and pharmaceutical applications of PNQs from *V. harmandiana* remain largely unexplored because of the lack of reference standards, as well as quantitative analytical methods.

NMR and MS are the major instruments commonly used in quantitative analysis of specialized metabolites in various types of samples. In natural product research, NMR is typically the recommended technique for structural determination and qualitative analysis, although the requirement for large amounts of sample remains a challenge with this technique. In contrast, MS provides greater sensitivity and is well suited for the high-throughput characterization of specialized metabolites in plant metabolomes. Liquid chromatography in combination with tandem MS (LC-MS/MS) operating in multiple reaction monitoring (MRM) mode is now widely accepted as the standard technique for quantitative metabolomics owing to its excellent sensitivity, specificity, and fast scanning rate (Zhou and Yin, 2016). Fragmentation using electrospray ionization (ESI) in combination with collision cells in MS/MS represents an important development in plant metabolomics (Kuwayama et al., 2009; Pozo et al., 2009; Zhao et al., 2011; De Vijlder et al., 2018; Kind et al., 2018). The ESI-MS/MS mass spectra provide important information about the molecular structures of known and unknown metabolites. In the past few years, much effort has been focused on creating database platforms based on ESI-MS/MS to identify known and unknown metabolites from various sources (Wang et al., 2016; Guijas et al., 2018).

The classes of PNQ and ATQ have been previously found in different natural resources such as plants, microorganisms, and insects, and some of the compounds have been shown to exhibit a wide range of interesting biological activities (Banks et al., 1976; Zhang et al., 1997; Agarwal et al., 2000; Donner, 2007). The increasing number of compounds discovered in these families suggests that additional compounds, natural sources, and therapeutic activities of PNQs and ATQs remain to be discovered. Therefore, in this study, we developed an MS-based methodology that will facilitate the discovery of PNQs and ATQs, as well as other compounds sharing similar structures, in different biological sources. The analytical protocol and methodology were implemented based on low- and high-resolution MS (HRMS), which allowed us to accurately identify and quantify six PNQ and two ATQ metabolites (Figures 1A–H) from five different parts of *V. harmandiana*: heartwood, wood, bark, root, and leaves. Additionally, as all PNQ standards are currently not commercially available, we provided ESI-MS/MS mass spectra of the PNQs spectra fragmented at different collision energies.

## Materials and Methods

### Chemical and Reagents

A stable isotope of salicylic acid-D_6_ (SAL-D_6_), purchased from Sigma–Aldrich (Singapore), was used as an internal standard (IS). LC-MS grade acetonitrile (ACN), methanol (MeOH), and isopropanol (IPA) were purchased from RCI Labscan (Thailand) and Fisher Chemical (United States). Formic acid was purchased from Fisher Chemical (United States). HPLC-quality water (H_2_O) was purified using a Milli-Q water system from Millipore (France).

### PNQ and ATQ Standards

The standards of PNQ-288A, PNQ-288B, PNQ-318A, PNQ-318B, PNQ-302, and PNQ-332 (Figures 1A–F) were previously isolated and purified from the heartwood of *V. harmandiana* (Rhamnaceae) (Panthong et al., 2020). Briefly, the air-dried heartwood (600 g) was soaked in 3 L of MeOH for 6 days (repeated three times). The crude MeOH-extract (ca. 35 g) was further purified with different solvent combinations (see the detailed protocol in Supplementary Figure S3). The fractions 4, 5, 6, and 8, which contained PNQ compounds were further purified with different solvent combinations, column chromatography, and preparative thin-layer chromatography. The ATQ-270 (emodin) and ATQ-284 (questin) standards (Figures 1G,H) were obtained from the isolation of bark extracts of *V*. *maingayi* (Rhamnaceae) (Leewanich, 2005). Briefly, dried *V. maingayi* (ca. 18.6 kg) was soaked in 70 L of MeOH for 60 days (repeated six times). The crude MeOH extract (ca. 390.4 g) was further isolated into 14 fractions (see the detailed protocol in Supplementary Figure S4) where fractions 8 and 10, which contained ATQ compounds, were further purified with similar techniques as those used to purify the PNQs. All PNQ and ATQ structures were confirmed by NMR spectrometry (Bruker DPX 300, Bruker Ascend^TM^ 400 MHz spectrometers and a Jeol NMR 400 MHz spectrometer) using tetramethylsilane as an IS. The method yielded the compounds with percent purity; PNQ-288A (93%), PNQ-288B (94%), PNQ-318A (97%), PNQ-318B (95%), PNQ-302 (97%), PNQ-332 (99%), ATQ-270 (74%), and ATQ-284 (91%).

### Plant Sampling and Sample Extraction

Heartwood, wood, bark, root, and leaves of *V. harmandiana* were collected from the Trang Province, Thailand, in March 2019 (lat. 7°47′12.8″ N, long. 99°30′55.0″ E; altitude 104 m a.s.l.). Because *V. harmandiana* is a rare species occurring only in the deep tropical rain forests of southern Thailand in a national park, obtaining biological replicates was restricted. Therefore, one biological replicate was reported, while the technical replicates were performed at least *n* = 3. After sampling, the samples were washed with tap water to remove dirt and dried in an oven at 80°C until a constant weight was obtained. The samples were ground into powder. Prior to extraction, 50 μL aliquot of 500 μM (0.025 μmol) SAL-D_6_ was added to a conical tube before adding 10 mg of powdered sample and 1 mL of MeOH. The mixture was extracted by using an ultrasonication bath at 60°C for 30 min. The extraction was repeated, and two crude extracts were combined. After the extraction, the crude extract was evaporated to dryness by a vacuum concentrator (Labconco, MO, United States) and redissolved in 1 mL of MeOH. Finally, the sample was filtered with hydrophilic polyvinylidene fluoride and diluted when necessary before LC-MS analysis. The pooled sample (solution of 10 mg/mL combined from each part of *V. harmandiana*) was analyzed periodically across the sample sequence to monitor instrumental variations.

#### Preparation of Standard Stock Solutions

The stock solutions were prepared by dissolving the standards in MeOH. The stock concentrations for all the standards were 500 μM, which corresponds to 0.14 mg/mL for PNQ-288A, PNQ-288B, ATQ-270, and ATQ-284; 0.16 mg/mL for PNQ-318A and PNQ-318B; 0.15 mg/mL for PNQ-302; and 0.17 mg/mL for PNQ-332. For IS, 500 μM SAL-D_6_ was prepared as a stock solution in MeOH (0.07 mg/mL).

#### Preparation of Calibration Standards

To carry out the standard addition procedure, two diluents were prepared for the calibration standard solutions. Diluent 1 was an individual sample matrix prepared by diluting each sample extract (heartwood, wood, bark, root, and leaves) 40 × with MeOH. Diluent 2 was a pooled-sample matrix prepared by mixing 1 mL of all diluent 1 solution of heartwood, wood, bark, root, and leaves. Calibration standard solutions used for spike recovery experiments were prepared using diluent 1 of each particular part of the plant as a solvent. Another set of calibration standard solutions used for the quantitative analysis of each part of *V. harmandiana* was prepared by using diluent 2 as a solvent.

### Quantitative Analysis of PNQs and Structure Elucidation by Ultraperformance Liquid Chromatography–Tandem Mass Spectrometry

Quantitative analyses were performed using an Acquity UPLC I-class system, coupled to a Waters Xevo TQ-S MS/MS equipped with an ESI source (Waters, MA, United States). The ESI source was operated in negative mode. Nitrogen (N_2_) and argon (Ar) were used as the desolvation and collision gases, respectively. MS conditions were as follows: capillary voltage at 1.50 kV, source offset at 60 V, desolvation gas (N_2_) with flow at 800 L/h at 550°C, source temperature at 150°C, cone gas flow (N_2_) at 150 L/h, nebulizer gas (N_2_) at 7.0 bar, and collision gas (Ar) at 0.15 mL/min. The MS conditions for each analyte were determined empirically via direct infusion of individual standard solution.

The UPLC-MRM/MS and other chromatographic conditions, modified from previous published protocols, were used for the quantitative analysis (Lin et al., 2018; Khoomrung et al., 2020). A 5-μL aliquot of standards or samples was injected into a reversed-phase column (Waters CSH C_18_, 2.1 × 100 mm, 1.7 μM column; MA, United States). The column temperature was set at 30°C with a flow rate of 0.4 mL/min. The weak and strong needle washes were 5% ACN in H_2_O (vol/vol) and 1:1:1:1 H_2_O/ACN/MEOH/IPA, respectively. The mobile phase comprised 0.1% formic acid (vol/vol) for mobile phase A and 100% ACN for mobile phase B. The chromatographic separation began with 1% B, and increased with linear gradient to 5% B over 1 min, to 20% B over 0.1 min, to 40% B in 2.9 min, to 50% over 5 min, to 60% over 1.5 min, to 80% over 1.5 min, and to 90% over 2 min. Finally, it was returned to 1% B at 15 min and held for 3 min with the total run time of 18 min. The optimized UPLC-MS/MS was applied to collect MS/MS spectra of individual analyte standards in a heartwood sample using collision energies of 10, 20, and 40 eV. The MS/MS spectra were acquired using the product ion scan mode.

For quantification, MRM transitions for each metabolite were determined (Supplementary Table S1 and Supplementary Figure S5). The concentrations of eight compounds in the *V. harmandiana* plant were quantified by the standard addition method. Eight calibration standard solutions were prepared by spiking known amounts of target analytes and SAL-D_6_ into a pooled-sample matrix (diluent 2) at concentrations ranging from 0.05 to 20 μM. We note that the quantification was performed using a single MRM transition for each analyte. However, we further validated the accuracy of the UPLC-MRM/MS method using the high-resolution UPLC coupled to high-resolution quadrupole and time-of-flight tandem MS (UPLC-Q-TOFMS).

### Accurate Mass Measurement by High-Resolution UPLC-Q-TOFMS

The accurate mass measurements were conducted on a Q-TOFMS (a Waters Acquity UPLC system coupled to a SYNAPT G2-Si HDMS, Waters, United States and United Kingdom) equipped with an ESI. Chromatographic separation was performed on a CSH C_18_ 2.1 × 100 mm, 1.7 μM column (Waters, MA, United States). The UPLC and MS conditions were similar to the analysis using the UPLC-MS/MS. The analysis was carried out in the MS^E^ full scan mode (multiparallel collision-induced dissociation) with 0.5 s scan time and collision energy ramp range of 20–45 eV. Mass calibration was performed with sodium formate for the range of *m/z* 50–1,200. Leucine enkephalin was acquired as a lockspray signal for acquisition correction of *m/z* during the operation. The 20 μM mix standard of eight compounds was injected to UPLC-ESI-Q-TOF to determine the accurate mass as shown in Supplementary Table S2. The MS analyses were performed in MS^E^ mode, where Function 1 with low collision energy was used to monitor the accurate mass of precursor ions, whereas Function 2 with high collision energy was used to monitor the accurate mass of product ions. All of spectral data were processed using MassLynx (v.4.1) software (Waters Corporation).

### Quality Assurance and Quality Control

#### Intraday and Interday Experiments

To evaluate the reproducibility of the analytical method, a standard mix solution was prepared for intraday and interday experiments. The intraday precision was determined by the evaluation of 10 replicates of the standard mix solution. The interday precision was determined by the evaluation of 10 replicates of standard mix solution for 3 consecutive days. Shown in Table 1, means and percent residual standard deviations (%RSD) were calculated based on peak area and retention time (RT) of each analyte. The %RSD values of peak area and RT for intraday measurement were < 3% and < 0.1%, respectively. For the interday experiment, except for PNQ288B, the %RSD values of peak area and RT were < 2.5% and < 0.3%, respectively (Table 1).

**TABLE 1:** Precision (intraday and interday), LOD, and LOQ of each compound with UPLC-ESI-MS/MS method.

Compound	5 μL Injected (ng/mL)	Peak area (%RSD)		Retention time (%RSD)		This study	
		Intraday (*n* = 10)	Interday^*a*^ (*n* = 3)	Intraday (*n* = 10)	Interday^*a*^ (*n* = 3)	LOD (ng/mL)	LOQ (ng/mL)
PNQ-288A	13.28	2.98	2.43	0.06	0.02	0.11	0.36
PNQ-288B	12.72	0.51	15.30	0.10	0.18	0.76	2.52
PNQ-318A	12.72	1.69	2.02	0.05	0.01	0.20	0.67
PNQ-318B	11.52	0.70	1.00	0.10	0.24	0.45	1.51
PNQ-302	11.52	0.64	0.70	0.09	0.02	0.39	1.31
PNQ-332	12.08	1.44	0.75	0.00	0.00	0.34	1.14
ATQ-270	10.80	2.20	1.36	0.05	0.02	0.24	0.79
ATQ-284	11.36	1.73	0.51	0.07	0.02	0.03	0.09

*^a^Interday precision was determined over the period of 3 days (10 measurements on each day).*

#### Plant Sample Analysis

The sample run sequence starts with the calibration standards (three injections for each concentration), solvent blank (methanol), pooled-sample matrix, plant samples, and a calibration standard. To evaluate the MS system suitability, the pooled-sample matrix was run with each batch. There was no significant deviation of responses among the pooled-sample matrix runs. The average percent recovery of IS in the samples was 82% ± 1% (Supplementary Table S3).

### Data Analysis

The acquired UPLC-MRM/MS data were exported to Microsoft Excel 2019 for data processing including quantification and statistical analysis. To generate calibration curves, the peak area of each analyte in the pooled-sample matrix was subtracted from the peak area of each analyte measured in the samples. Relative responses were calculated as (average peak area of an analyte × IS concentration)/average peak area of IS. Then, the relative responses were plotted with respect to concentrations for each analyte. All the calibration curves yielded a good linear correlation, *R*^2^ > 0.99, with at least three calibration data points (Supplementary Table S4). The derived linear calibration equations were used to quantify analyte concentrations in the samples.

The limit of detection (LOD) and limit of quantification (LOQ) were calculated using the following equations, LOD = 3 × SD/*m* and LOQ = 10 × SD/*m*, respectively, where SD, standard deviation derived from three injections of the lowest analyte concentration; and *m*, slope of the calibration curve (Khoomrung et al., 2013). The LOD and LOQ values are given in Table 1.

## Results and Discussion

### PNQ and ATQ Standards

PNQs were recently discovered in *V. harmandiana*, but information regarding their chromatographic behaviors and MS responses remains limited (Panthong et al., 2020). Given the structural similarity of PNQs to ATQs, we used ATQ-270 (emodin) and ATQ-284 (questin) as positive controls to observe all necessary chemical and MS behaviors during the analytical method development. The PNQ and ATQ standards in the present study were isolated from the heartwood extracts of *V. harmandiana* extracts and the bark extracts of *V. maingayi* (Leewanich, 2005; Panthong et al., 2020). Subsequently, the structures of each purified standard were confirmed by NMR spectroscopy, followed by HRMS measurements with mass accuracy (Δ*m*) of 1.1–6.6 ppm and mass resolving power of 17,000–30,000 (at full width at half height; see Supplementary Table S2).

### MS Detection and Chromatographic Separation of PNQ and ATQ Metabolites

To optimize the MS/MS settings for each analyte (i.e., precursor and product ions, cone voltage, and collision energy), individual standards were directly infused into the MS/MS operated in both POS and NEG modes. All the standards exhibited much better signal responses in NEG mode (Supplementary Figure S6); therefore, the quantitative analysis was conducted only in NEG mode. The stable isotope of SAL-D_6_ (MW 144.16) used as the IS was detected with the molecular ion of *m/z* 141 (NEG). The MRM transition of *m/z* 141 > 97 represents the cleavage of a CO_2_ molecule (Supplementary Figure S7). All the MRM transitions and MS/MS conditions for the quantitative analysis are summarized in Supplementary Table S1. The chromatographic separation was achieved using a reversed-phase C_18_ column. To reduce the high back pressure of the UPLC during the chromatographic run, we used ACN as a mobile phase instead of MeOH. Our experiments showed that using MeOH with the flow rate of 0.3 mL/min caused a back pressure greater than 12,000 psi, which prevented operation beyond this point. However, using ACN at the same flow rate reduced the back pressure to less than 7,000 psi. This allowed the system to increase the flow rate to 0.4 mL/min, while maintaining the back pressure ∼9,000 psi. For the mobile phase A (H_2_O), we introduced 0.1% formic acid as a modifier to keep the pH at ∼3, which was suitable for the separation. This was shown to improve the separation performance by reducing peak tailing of PNQs and ATQs. Finally, we obtained the optimal gradient program with a total run time of 18 min to quantify six PNQs, two ATQs, and the IS (Supplementary Table S1 and Supplementary Figure S5). Our developed method performs very well in terms of precision and accuracy, with percent relative standard deviation values (%RSD) of less than 3%, calculated based on peak area and RT (Table 1). The LOD and LOQ, which were calculated based on the lowest standard concentration of each analyte (Khoomrung et al., 2013), were 0.03–0.76 and 0.09–2.52 ng/mL, respectively (Table 1). It was quite challenging to evaluate the quality of the method because the PNQs are relatively novel and have not been previously quantified. For the ATQs, although they are known compounds, the plant materials and analytical methods used by other studies differ from our study. Nonetheless, Wang et al. (2015) reported LOD and LOQ of 1 and 2 ng/mL, respectively, for ATQ-270 (emodin) in rhubarb using a similar technique, which are 5 and 2.5 times higher than those derived from our method (Table 1).

### Identification of PNQs and ATQ in *V. harmandiana*

**FIGURE 2:**
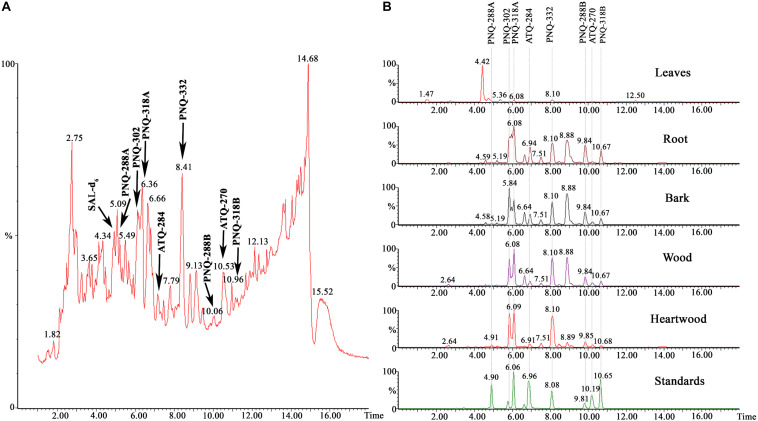
**(A)** A UPLC-MS chromatogram of a heartwood extract in MS full scan mode collecting *m/z* from 50 to 1,200. **(B)** UPLC-MRM/MS chromatograms of all target metabolites from different parts of *V. harmandiana*.

We applied our method to identify PNQ and ATQ metabolites in *V. harmandiana.* Based on the comparison of RTs and MRM transitions to those of the authentic standards, detection of these metabolites in *V. harmandiana* samples was achieved. The percent accuracy of RT performed on the UPLC-MS/MS and the high-resolution UPLC-Q-TOFMS ranged from 99.8% to 100.2% and from 99.8% to 100.2%, respectively. Mass accuracy (Δ*m*) derived from the measurements using the high-resolution Q-TOFMS ranged from 1.0 to 6.0 ppm (Supplementary Table S5). Because of the inherently complex matrix associated with plant extract, the additional RT comparison against the high-resolution UPLC-Q-TOFMS (Wu et al., 2020) was performed to validate the results obtained from the UPLC-MS/MS. Additionally, the ESI-MS/MS (POS and NEG) was performed at 10, 20, and 40 eV to collect MS/MS spectra of the analytes in standard solutions and *V. harmandiana* extracts using the UPLC-MS/MS. The results showed that MS/MS spectra of the target metabolites in the *V. harmandiana* were identical or similar to the reference spectra (Supplementary Figures S8: A–P). Figure 2A shows the identification of all PNQ and ATQ compounds found in heartwood extract in MS full scan mode at *m/z* 50–1,200 along with MRM transitions of all the target metabolites from different parts of *V. harmandiana* (Figure 2B). Overall, we were able to detect and identify six PNQs and two ATQs from *V. harmandiana.* Providing the MS/MS spectra of these metabolites in a database will facilitate the fast screening of PNQs and other quinone metabolites from other sources.

### ESI-MS/MS Structural Elucidation of PNQ Metabolites

**FIGURE 3:**
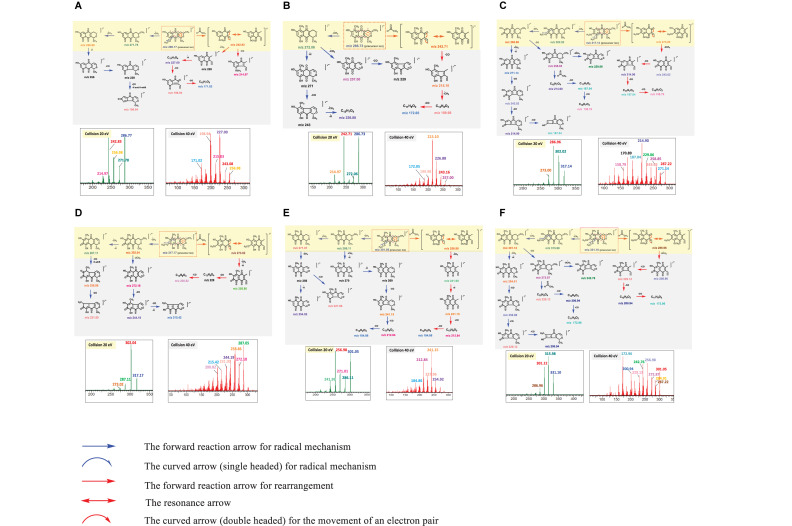
MS/MS spectra at 20 and 40 eV and structural elucidation for the target analytes (**(A)**, PNQ-288A; **(B)**, PNQ-288B; **(C)**, PNQ-318A; **(D)**, PNQ-318B; **(E)**, PNQ-302; **(F)**, PNQ-332).

An MS/MS spectrum obtained from a single collision energy failed to capture the full fragmentation information. For example, MS/MS spectrum of PNQ fragmentation at 20 eV yielded only the precursor ion and a few intense fragmentation ions, showing a loss of methyl radical [M-CH_3_]^–⋅^ or carbonyl [M-CO]^–⋅^ groups. Determination of the complete fragmentation pathways of PNQ metabolites therefore required MS/MS spectra obtained from application of more than one collision energy. Our experiments showed that the use of high energy at 40 eV generated a greater number of smaller fragmentation ions with the absence of precursor ions. This combined with the low collision energy (20 eV) MS/MS spectrum provided extensive information, allowing for the construction of the fragmentation pathways for all PNQs. The proposed ESI-MS/MS fragmentation pathways of individual PNQs provided below were derived from the MS/MS experiments using highly purified PNQ standards, which should be beneficial for the natural product research community in the aspect of prediction and identification of the PNQs or other quinone compounds in biological samples (Figure 3).

#### PNQ-288A

NEG ESI produced a molecular ion of PNQ 288A at *m/z* 286.77 [M-H]^–⋅^, with many potential pathways for molecular fragmentation (Figure 3A). In the first proposed pathway (blue arrow), the methyl radical [M-CH_3_]^–⋅^ at C_9_ of the methoxy group was eliminated to generate ions at *m/z* 271.78, after which a methyl radical [M-CH_3_]^–⋅^ at C_3_ was lost to yield an ion at *m/z* 256.98. The *m/z* 256.98 ion could lose a proton radical [M-H]^–⋅^ to yield an ion at *m/z* 256, and further lose two carbonyl groups [M-CO]^–⋅^ and a proton radical [M-H]^–⋅^ to result in an ion at *m/z* 198.94. Alternatively, the ion at *m/z* 286.77 could undergo rearrangement and lose an acetaldehyde group [M-C_2_H_4_O]^–⋅^ (red arrow) to generate an ion at *m/z* 242.83 (base peak) and further lose a carbonyl group [M-CO]^–⋅^ to yield an ion at *m/z* 214.95. In addition, an ion at *m/z* 242.83 could lose a methyl radical [M-CH_3_]^–⋅^, proton radical [M-H]^–⋅^, and two carbonyl groups 2[M-CO]^–⋅^ to yield ions at *m/z* 227.00, 198.94, and 171.02.

#### PNQ-288B

A molecular ion of compound PNQ-288B was produced at *m/z* 286.73 [M-H]^–⋅^ in negative mode ESI (Figure 3B). One potential fragmentation pathway involves the loss of methyl radical [M-CH_3_]^–⋅^ (blue arrow) at C_3_ to yield an ion at *m/z* 272.06. The *m/z* 272.06 ion could lose a methyl radical [M-CH_3_]^–⋅^ at C_1_ to yield an ion at *m/z* 257.00 or lose a proton radical [M-H]^–⋅^ and carbonyl group [M-CO]^–⋅^ together with a methyl radical [M-CH_3_]^–⋅^ and proton radical [M-H]^–⋅^ to result in an ion at *m/z* 226.88. In an alternative pathway, the ion at *m/z* 286.73 could first lose an acetaldehyde group [M-C_2_H_4_O]^–⋅^ by the rearrangement to generate an ion at *m/z* 242.71 (base peak) and further lose a carbonyl group [M-CO]^–⋅^ to yield an ion at *m/z* 215.10. In addition, the *m/z* 215.10 ion could lose a methyl radical [M-CH_3_]^–⋅^ and carbonyl group [M-CO]^–⋅^ to yield ions at *m/z* 199.98 and 172.05, respectively.

#### PNQ-318A

NEG ESI generated a molecular ion of compound PNQ-318A at *m/z* 317.14 [M-H]^–⋅^. Illustrated in Figure 3C, the first proposed pathway (blue arrow) of molecular fragmentation begins with the elimination of two methyl radicals 2[M-CH_3_]^–⋅^ at C_9_ and C_8_ of the two methoxy groups to yield ions at *m/z* 302.02 and 286.96 (base peak), respectively. An ion at *m/z* 286.96 could lose a methyl radical [M-CH_3_]^–⋅^ and proton radicals [M-H]^–⋅^ to generate an ion at *m/z* 271.14, which could then lose three carbonyl groups 3[M-CO]^–⋅^ to yield ions at *m/z* 243.02, 214.90, and 187.04. Alternatively, an ion at *m/z* 286.96 could lose a carbonyl group [M-CO]^–⋅^ to yield an ion at *m/z* 258.85. The *m/z* 258.85 ion could then lose two methyl radicals 2[M-CH_3_]^–⋅^ to generate an ion at *m/z* 229.06 or lose an acetaldehyde group [M-C_2_H_4_O]^–⋅^ by the rearrangement and lose two carbonyl groups 2[M-CO]^–⋅^ to yield ions at *m/z* 214.90, 187.04, and 158.79, respectively. In yet another potential pathway, the ion at *m/z* 317.14 could first be rearranged (red arrow) and lose an acetaldehyde group [M-C_2_H_4_O]^–⋅^ to generate an ion at m/z 273.00 and further lose two methyl radicals 2[M-CH_3_]^–⋅^ and three carbonyl groups 3[M-CO]^–⋅^ to yield ions at *m/z* 243.02, 214.90, 187.04, and 158.79.

#### PNQ-318B

NEG ESI produced a molecular ion of compound PNQ-318B at *m/z* 317.17 [M-H]^–⋅^. Illustrated in Figure 3D, fragmentation could have initiated with the loss of a methyl radical [M-CH_3_]^–⋅^ at C_8_ of the methoxy group (blue arrow) to generate an ion at *m/z* 302.04 (base peak). The ion at *m/z* 302.04 could lose a methyl radical [M-CH_3_]^–⋅^ and proton radicals [M-H]^–⋅^ to produce an ion at *m/z* 287.11, followed by the loss of two carbonyl groups 2[M-CO]^–⋅^ to yield ions at *m/z* 258.86 and 231.00, respectively. Alternatively, the ion at m/z 302.04 could lose two methyl radicals 2[M-CH_3_]^–⋅^ and two carbonyl groups 2[M-CO]^–⋅^ and a proton radical [M-H]^–⋅^ to yield ions at m/z 272.18, 244.19, and 215.42, respectively. Another potential fragmentation pathway of the *m/z* 317.17 ion (red arrow) begins with a rearrangement and loss of an acetaldehyde group [M-C_2_H_4_O]^–⋅^ to generate an ion at *m/z* 273.02, after which the loss of a methyl radical [M-CH_3_]^–⋅^, proton radical [M-H]^–⋅^, and two carbonyl groups 2[M-CO]^–⋅^ yields ions at *m/z* 256.86 and 200.82.

#### PNQ-302

A molecular ion of compound PNQ-302 was generated at *m/z* 301.05 [M-H]^–⋅^ in negative mode ESI. The first proposed pathway of the *m/z* 301.05 ion fragmentation begins with the elimination of a methyl radical [M-CH_3_]^–⋅^ at C_9_ of the methoxy group (blue arrow) to generate an ion at *m/z* 286.11 (Figure 3E). The *m/z* 286.11 ion could then lose a methyl radical [M-CH_3_]^–⋅^ to yield an ion at *m/z* 271.01 or instead lose a methyl radical [M-CH_3_]^–⋅^ and two proton radicals 2[M-H]^–⋅^ to yield an ion at *m/z* 269. The *m/z* 269 ion subsequently eliminates three carbonyl groups 3[M-CO]^–⋅^ to generate ions at *m/z* 241.15, 212.84, and 184.86, respectively. After the ion at 271.01 loses a methyl radical [M-CH_3_]^–⋅^, it could lose a proton radical [M-H]^–⋅^ or a carbonyl group [M-CO]^–⋅^ to yield ions at *m/z* 254.92 or *m/z* 227.96, respectively. In an alternative fragmentation pathway (red arrow), the ion at *m/z* 301.05 is first rearranged and loses an acetaldehyde group [M-C_2_H_4_O]^–⋅^ to generate an ion at *m/z* 256.93 (base peak) and further loses a methyl radical [M-CH_3_]^–⋅^, proton radical [M-H]^–⋅^, and two carbonyl groups 2[M-CO]^–⋅^ to yield ions at *m/z* 241.99, 241.15, 212.84, and 184.86.

#### PNQ-332

NEG ESI produced a molecular ion of compound PNQ-332 at *m/z* 331.10 [M-H]^–⋅^. One potential fragmentation pathway (blue arrow) begins with the loss of two methyl radicals 2[M-CH_3_]^–⋅^ at C_9_ and C_8_ of the two methoxy groups to generate ions at *m/z* 315.98 (base peak) and 301.12, respectively (Figure 3F). The ion at *m/z* 301.12 could lose a methyl radical [M-CH_3_]^–⋅^ and proton radical [M-H]^–⋅^ to yield an ion at *m/z* 284.91, after which the elimination of three carbonyl groups 3[M-CO]^–⋅^ generates ions at *m/z* 256.98, 229.12, and 200.94. The *m/z* 301.12 ion could alternatively lose a carbonyl group [M-CO]^–⋅^ to yield an ion at *m/z* 272.87, followed by the loss of either two methyl radicals 2[M-CH_3_]^–⋅^ to yield an ion at *m/z* 242.76 or the loss of an acetaldehyde group [M-C_2_H_4_O]^–⋅^ by rearrangement and two carbonyl groups 2[M-CO]^–⋅^ to generate ions at *m/z* 229.12, 200.94, and 172.96, respectively. In another potential pathway (red arrow), the ion at *m/z* 331.10 might be first be rearranged and lose an acetaldehyde group [M-C_2_H_4_O]^–⋅^ to generate an ion at *m/z* 287.96 and further lose two methyl radicals 2[M-CH_3_]^–⋅^ and three carbonyl groups 3[M-CO]^–⋅^ to yield ions at *m/z* 256.98, 229.12, 200.94, and 172.96, respectively.

**FIGURE 4:**
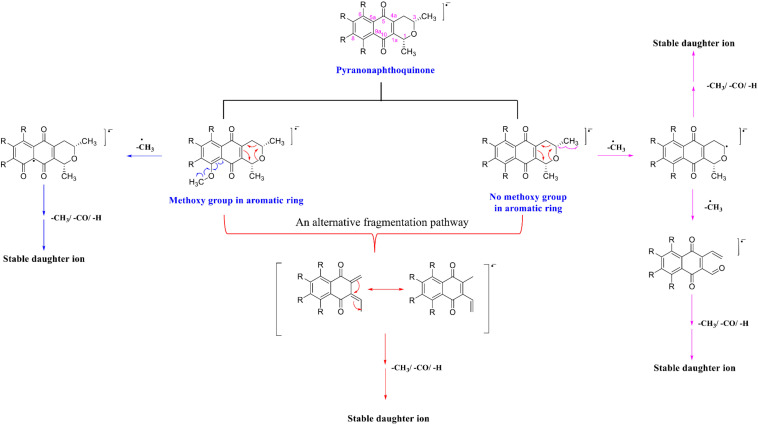
A generalized fragmentation mechanism for the target metabolites.

In summary, the fragmentation mechanism of all PNQs by NEG ESI-MS/MS likely followed one of product two pathways (Figure 4). In the first pathway, if the PNQ molecule contains an aromatic ring with a methoxy group, the methyl radical of the methoxy group will be eliminated to generate a keto group. On the other hand, if there is no methoxy group on an aromatic ring, the methyl group on the pyrano ring will be eliminated instead. It may successively lose a methyl radical [M-CH_3_]^–⋅^, carbonyl group [M-CO]^–⋅^, or H-shift to generate a stable product ion. In an alternative fragmentation pathway, the precursor ion could undergo rearrangement by loss of an acetaldehyde group [M-C_2_H_4_O]^–⋅^ and may then successively lose a methyl radical [M-CH_3_]^–⋅^, carbonyl group [M-CO]^–⋅^, or H-shift to generate a stable product ion.

### Absolute Quantification of PNQs and ATQs in *V. harmandiana*

**FIGURE 5:**
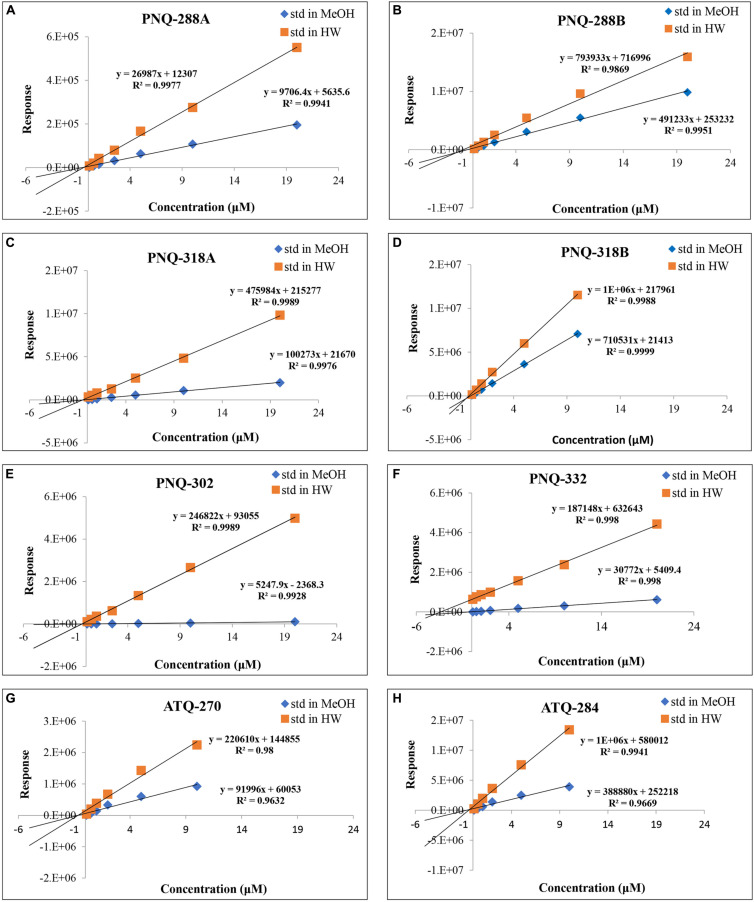
Comparison of calibration curves for all the target analytes prepared in MeOH vs individual sample matrix (standard addition method) of PNQ-288A **(A)**, PNQ-288B **(B)**, PNQ-318A **(C)**, PNQ-318B **(D)**, PNQ-302 **(E)**, PNQ-332 **(F)**, ATQ-270 **(G)** and ATQ-284 **(H)**.

Typically, an external calibration curve prepared in a pure solvent is the conventional method and broadly used in quantifying metabolites in various types of biological samples (Khoomrung et al., 2012, 2014; Tippmann et al., 2016). Because of the large number of metabolites in plant extracts, this often causes ion suppression or enhancement that subsequently yields erroneous results (Furey et al., 2013). We addressed this issue by creating a calibration curve for each PNQ standard prepared in MeOH (standard matrix) and compared with the calibration curve that was prepared in the sample matrix (matrix matching) (Panuwet et al., 2016). We evaluated the matrix effect through the regression between concentrations and peak responses of each metabolite. The differences in slope of each calibration between the standard matrix (MeOH) and the sample matrix lines were compared. The results in Figure 5 show that there were matrix effects in all cases. This impacted the accuracy of the method as observed in the percent recoveries of SAL-D_6_ from the spiking experiments (quantified from calibration curves that prepared in MeOH), which were only 0.6–4% (*n* = 3; Supplementary Table S6). In contrast, the percent recoveries of SAL-D_6_ improved to 84–128% (*n* = 3; Supplementary Table S6) when the quantification was performed in a pooled-sample matrix. In general, matrix effect is a common problem in MS techniques when dealing with plants, blood, urine, and animal tissue. Several strategies have been proposed to overcome this problem, e.g., selective extraction, sample clean-up, external calibration using matrix-matched samples, standard addition, IS, and dilution of sample extract (Furey et al., 2013; Panuwet et al., 2016; Tippmann et al., 2016). To reduce the sample preparation time and maintain high accuracy, we used a combination of a standard addition and IS for quantitative determination of PNQs and ATQs in *V. harmandiana.*

**FIGURE 6:**
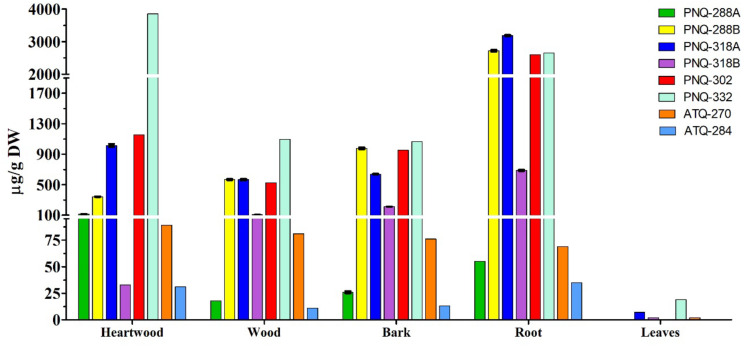
Concentrations of six PNQs and two ATQs from five different parts of *V. harmandiana*. Note that the results are based on one biological replicate, and the precision is calculated from three technical replicates.

The optimized protocol was then applied to quantify PNQ and ATQ metabolites from *V. harmandiana.* The percent recovery of the protocol was evaluated by spiking SAL-D_6_ in all the samples. The average percent recovery was 82% ± 1% (*n* = 15; Supplementary Table S3). To our knowledge, there has not been a study reporting the concentrations of the PNQs. This is partly because the authentic standards are not commercially available. However, to place our percent recovery into a broader context, the percent recovery reported by other studies using UPLC-MS/MS to quantify phytochemicals ranges from 90% to 103% (Ertaş et al., 2014; Kumar et al., 2018). The good percent recovery of IS from the spiking experiments supports the accuracy and reliability of the method. The highest concentrations of PNQs were primarily observed in the root of *V. harmandiana*, where concentrations of PNQ-288B, 318A, 302, and 332 ranged from 2,597 to 3,185 μg/g dry weight, although PNQ-332 was found at greater concentrations in the heartwood at 3,856 ± 29 μg/g. These same PNQs (288B, 318A, 302, and 332) were generally abundant in the heartwood, wood, and bark, with concentrations of each exceeding 300 μg/g, but PNQ-288A, 318B, and the ATQs were relatively low in abundance. Unlike the PNQs, the abundance of the ATQs was more constant across the different plant parts, ranging from 11 to 89 μg/g, except for the leaves. The leaves of *V. harmandiana* exhibited by far the lowest PNQ and ATQ concentrations among the plant parts, where some were below the LOD (Figure 6 and Supplementary Table S3).

## Conclusion

In summary, natural products such as PNQs possess a diverse range of therapeutic potential, but the inability to properly quantify their abundance within plants, insects, or other biological sources hinders investigative efforts. We developed and validated a UPLC-ESI-MRM/MS method to quantify the absolute abundance of six different PNQs in *V. harmandiana* heartwood, wood, bark, root, and leaves. As PNQs have a rigid fused three-ring backbone, it was required to combine fragmentation patterns derived from two collision energies of 20 and 40 eV to obtain the complete MS/MS fragmentation pathways of PNQs. This highlighted the importance of additional mass spectral information to elucidate fragmentation pathways of specialized metabolites presented in natural products, which generally have more complex chemical structures than other small molecules. The plant extracts generally contain many components that can interfere with the accuracy of the quantification process. We have shown to that such problem can be overcome by classical standard addition and the addition of a stable isotope IS. The results contributed to a more quantitative understanding of PNQs and their distribution in *V. harmandiana*. We expect our method will help support and accelerate future quantitative natural product research.

We note the requirement of at least three independent biological replicates for the quantitative analysis. However, the studied plant, *V. harmandiana*, is a rare species occurring only in the deep tropical rain forests of southern Thailand in a national park in which plant sample collection can be restricted. We made our best effort to follow the national park’s restriction and, at the same time, conduct high-quality research. Thus, we harvested one plant, but carried out experiments using three technical replicates, which still allows us to provide useful information for comparison across species and for our future research on medicinal properties of this plant. Another study limitation was the choice of IS. The ideal IS compounds are isotopically labeled analogs of the target compounds. However, because of the lack of commercially available standards and our limited resources, SAL-D_6_ was alternatively chosen as it at least contains an aromatic ring. Nonetheless, we deliberately developed the quantitative method that could minimize other source of greater errors, such as matrix effect. Although the uncertainty due to the use of SAL-D_6_ inevitably exists, we believe that it did not significantly affect the overall results.

## Data Availability Statement

The original contributions presented in the study are included in the article/Supplementary Materials, further inquiries can be directed to the corresponding author/s.

## Author Contributions

SL performed the experiments, analyzed the data, prepared figures, wrote the manuscript, and approved the manuscript. KK performed the experiments, analyzed the data, and approved the manuscript. NJ supervised the study, analyzed the data, wrote and approved the manuscript. SH and NN prepared the sampling and sample and approved the manuscript. JR analyzed the data, wrote the manuscript, and approved the manuscript. IN analyzed the data and approved the manuscript. YS resourced the study, supervised the study, and approved the manuscript. CK supervised the study, analyzed the data, and approved the manuscript. VR supervised the study, sampling, and approved the manuscript. SK designed and supervised the study, sampling, analyzed the data, wrote the manuscript, and approved the manuscript. All authors contributed to the article and approved the submitted version.

## Conflict of Interest

The authors declare that the research was conducted in the absence of any commercial or financial relationships that could be construed as a potential conflict of interest.
