# Supplementary material for: UPLC-ESI-MRM/MS for Absolute Quantification and MS/MS Structural Elucidation of Six Specialized Pyranonaphthoquinone Metabolites From Ventilago harmandiana
Source: Front Plant Sci. 2021 Jan 11;11:602993. doi: 10.3389/fpls.2020.602993 (PMC7830255; doi:10.3389/fpls.2020.602993)
Supplement: Supplementary file 1 [file Data_Sheet_1.docx]

**UPLC-ESI-MRM/MS for absolute quantification and MS/MS structural elucidation of six specialized pyranonaphthoquinone metabolites from *Ventilago harmandiana***

Suphitcha Limjiasahapong^1,2,3^, Khwanta Kaewnarin^1,2^, Narumol Jariyasopit^1,2^, Sakchai Hongthong^3,4^, Narong Nuntasaen^5^, Jonathan L. Robinson^6^, Intawat Nookaew^7^, Yongyut Sirivatanauksorn^2^, Chutima Kuhakarn^3^, Vichai Reutrakul^3^, Sakda Khoomrung^1,2,3,*^

^1^Metabolomics and Systems Biology, Department of Biochemistry, Faculty of Medicine Siriraj Hospital, Mahidol University, Bangkok 10700, Thailand

^2^Siriraj Metabolomics and Phenomics Center, Faculty of Medicine Siriraj Hospital, Mahidol University, Bangkok 10700, Thailand

^3^Center of Excellence for Innovation in Chemistry (PERCH-CIC), Faculty of Science, Mahidol University, Rama 6 Road, Bangkok 10400, Thailand

^4^Division of Chemistry, Faculty of Science and Technology, Rajabhat Rajanagarindra University, Chachoengsao, 24000, Thailand

^5^The Forest Herbarium National Park, Wildlife and Plant Conservation Department, Ministry of Natural Resources and Environment, Thailand

^6^Department of Biology and Biological Engineering, National Bioinformatics Infrastructure Sweden, Science for Life Laboratory, Chalmers University of Technology, Kemivägen 10, SE-41258 Gothenburg, Sweden

^7^Department of Biomedical Informatics, College of Medicine, University of

Arkansas for Medical Sciences, Little Rock, AR 72205, USA

***Corresponding author:** Sakda Khoomrung, phone + 662-419-5506

sakda.kho@mahidol.edu

**Keywords:** Mass spectrometry, Targeted metabolite profiling, Specialized metabolites, Structural elucidation, Natural products


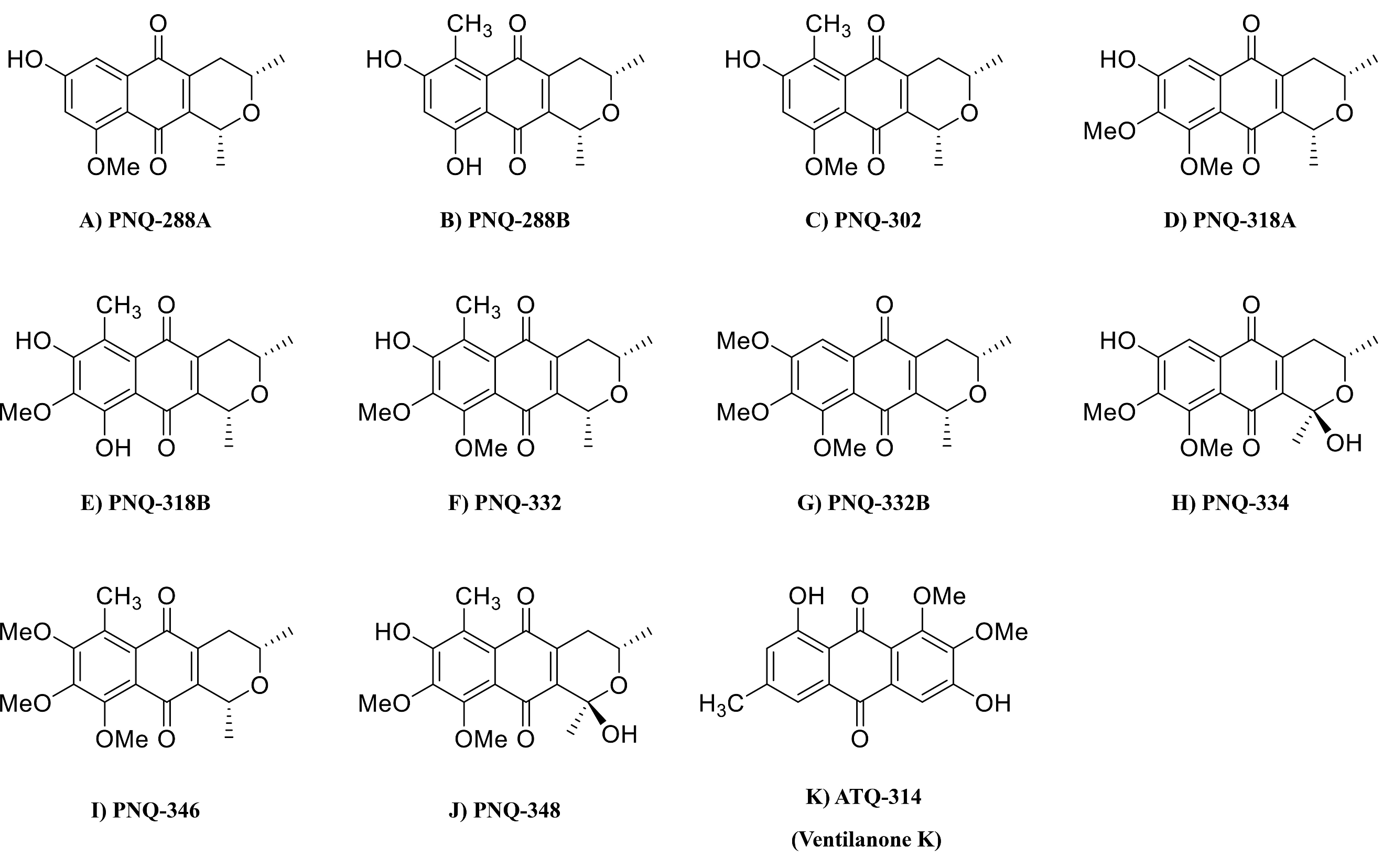


**Fig. S1** Chemical structures of novel pyranonaphthoquinone (PNQ) and anthraquinone (ATQ) metabolites identified from *V. harmandiana*.


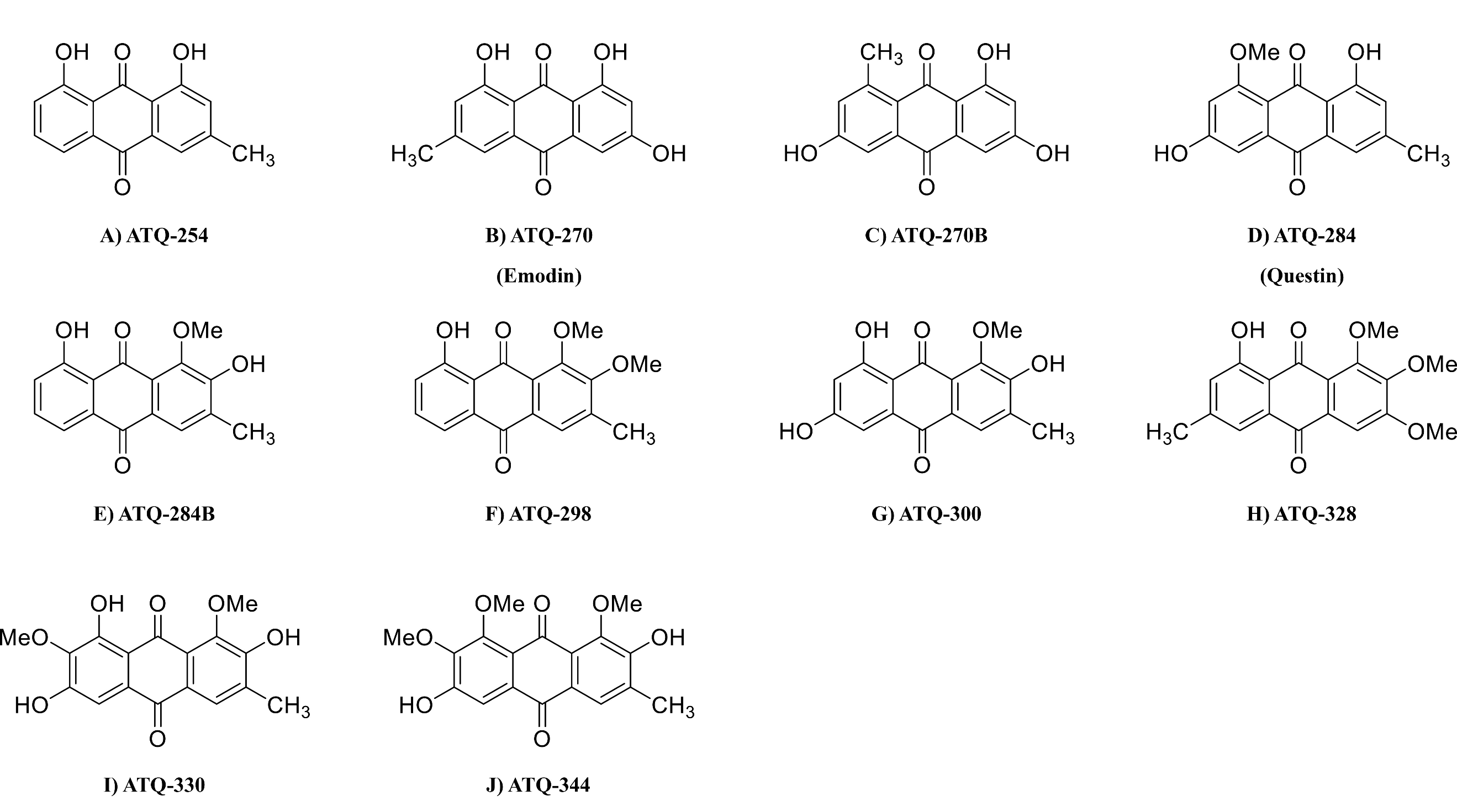


**Fig. S2** Chemical structures of known anthraquinone (ATQ) metabolites identified from *Ventilago* genus.


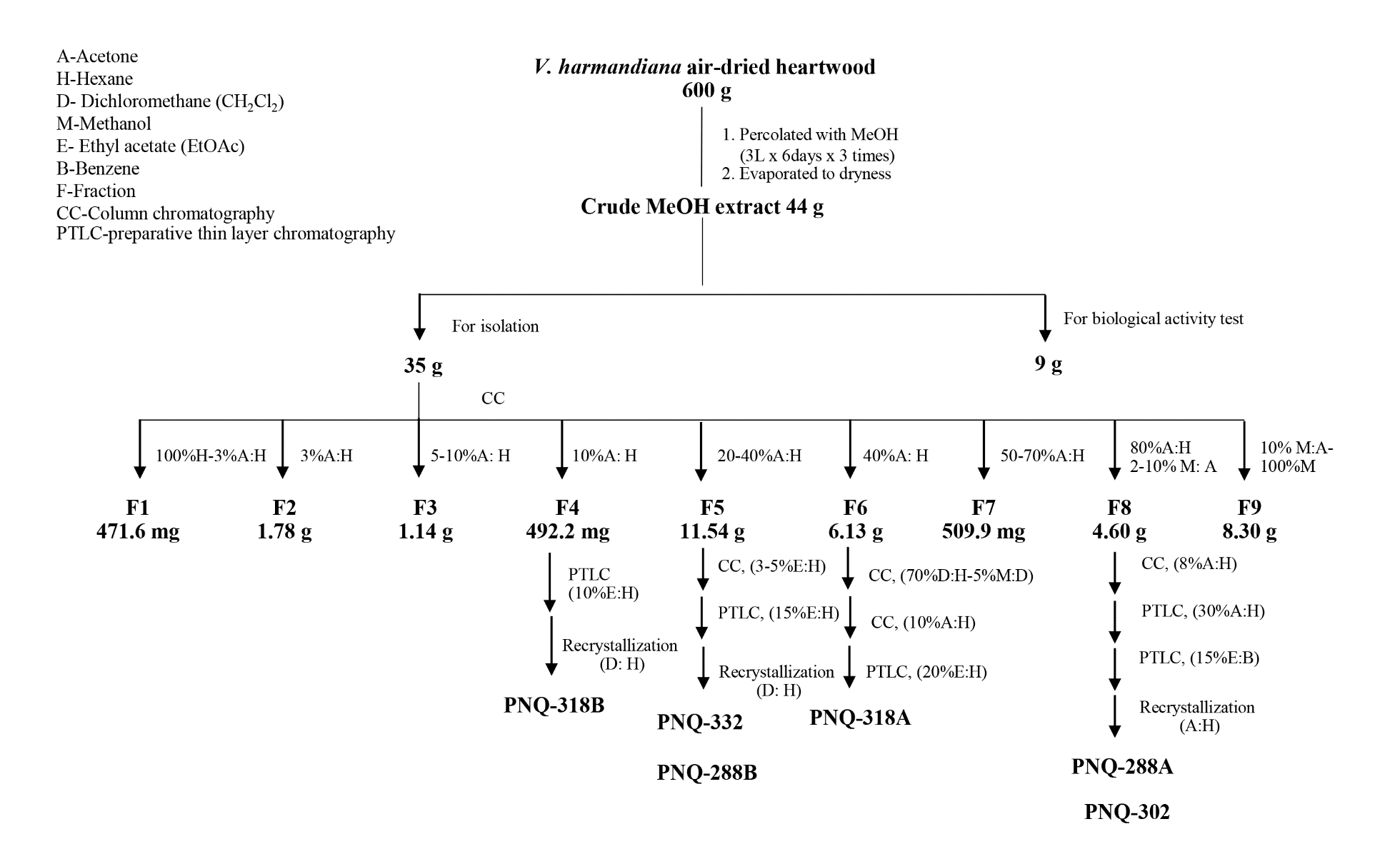


**Fig. S3** Protocol for the extraction and isolation of PNQ standards (data from Panthong et al., 2020).


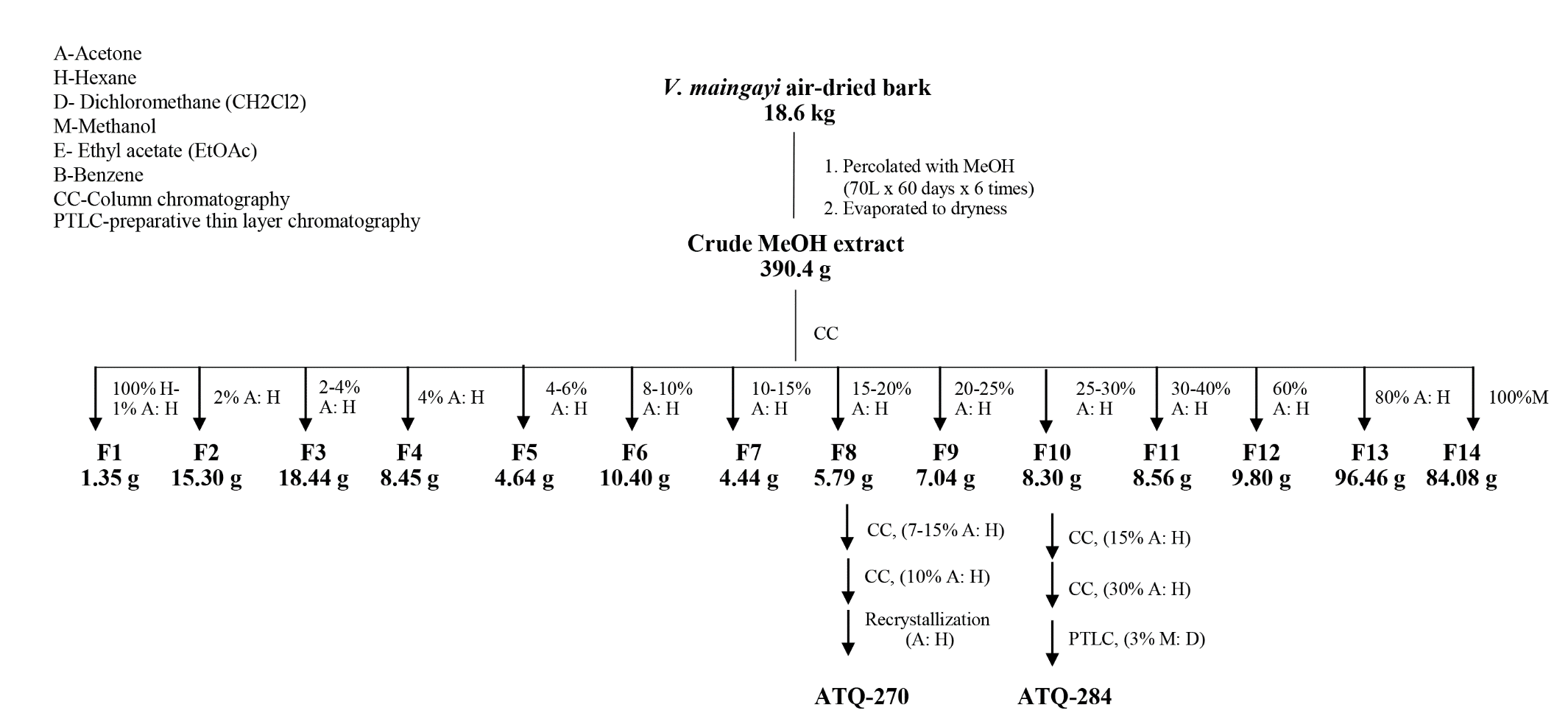


**Fig. S4** Protocol for the isolation of ATQ standards (data from Leewanich, 2005).

**Table S1** Summary MRM measurement conditions of PNQs, ATQs and IS (NEG).

| No. | Compound | Precursor ion (m/z) | Product ion (m/z) | cone voltage (V) | collision energy (eV) | Retention time (min) |
| --- | --- | --- | --- | --- | --- | --- |
| 1 | PNQ-288A | 287 | 243 | 20 | 30 | 4.90 |
| 2 | PNQ-288B | 287 | 243 | 10 | 25 | 9.81 |
| 3 | PNQ-318A | 317 | 287 | 10 | 20 | 6.06 |
| 4 | PNQ-318B | 317 | 302 | 10 | 22 | 10.65 |
| 5 | PNQ-302 | 301 | 257 | 10 | 20 | 5.84 |
| 5 | PNQ-332 | 331 | 301 | 20 | 22 | 8.08 |
| 6 | ATQ-270 | 269 | 225 | 10 | 30 | 10.19 |
| 7 | ATQ-284 | 283 | 240 | 10 | 25 | 6.86 |
| 8 | *SAL-D_6_  (MW=144) | 141 | 97 | 15 | 15 | 4.71 |

*SAL-D_6_ = Internal standard (IS)


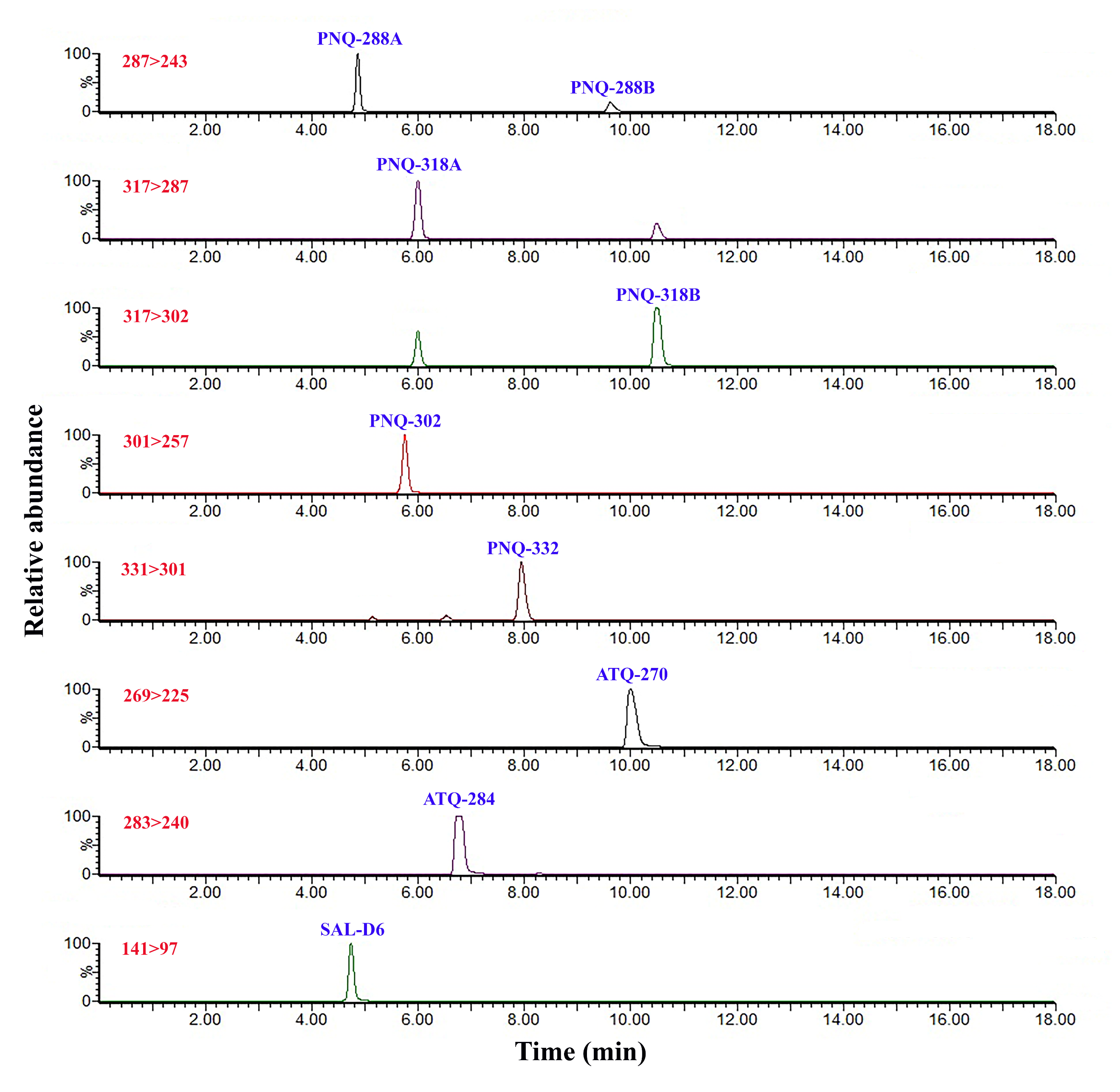


**Fig. S5** Extracted-ion chromatograms (XICs) of mix standard (PNQs, ATQs and IS).

**Table S2** High-resolution UPLC-QTOF-MS measurement of PNQ and ATQ standards.

| **Compounds** | **Molecular weight** | **Rt (min)** | **Calculated m/z** | **Measured m/z** | **Mass accuracy (∆m, ppm)** | **Mass Resolving power at FWHM** |
| --- | --- | --- | --- | --- | --- | --- |
| PNQ-288A | 288.0998 | 4.79 | 287.0919 | 287.0938 | 6.6 | 17000 |
| PNQ-288B | 288.0998 | 9.50 | 287.0919 | 287.0938 | 6.6 | 24000 |
| PNQ-318A | 318.1103 | 5.91 | 317.1025 | 317.1044 | 6.0 | 30000 |
| PNQ-318B | 318.1103 | 10.43 | 317.1025 | 317.1044 | 6.0 | 29000 |
| PNQ-302 | 302.1154 | 5.68 | 301.1076 | 301.1095 | 6.3 | 30000 |
| PNQ-332 | 332.1260 | 7.87 | 331.1182 | 331.1197 | 4.5 | 30000 |
| ATQ-270 | 270.0528 | 9.94 | 269.0450 | 269.0453 | 1.1 | 27000 |
| ATQ-284 | 284.0685 | 6.67 | 283.0606 | 283.0594 | -4.2 | 28000 |

**Table S3** Concentrations of six PNQs and two ATQs from five different parts of *V. harmandiana.* Noting that the results are based on one biological replicate, and the precision is calculated from three technical replicates.

| **Compounds** | **Concentration**  **µg/g dry weight (N=3)** | | | | | |
| --- | --- | --- | --- | --- | --- | --- |
|  | **Heartwood** | **Wood** | **Bark** | | **Root** | **Leaves** |
|  | µg/g±SD (%RSD) | µg/g±SD (%RSD) | µg/g±SD (%RSD) | | µg/g±SD (%RSD) | µg/g±SD (%RSD) |
| PNQ-288A | 113±2 (2) | 18±0 (2) | 26±1 (2) | | 55±0 (1) | N.D. |
| PNQ-288B | 342±3 (1) | 568±7 (1) | 977±11 (1) | | 2,722±26 (1) | N.D. |
| PNQ-318A | 1,015±18 (2) | 566±10 (2) | 639±5 (1) | | 3,185±19 (1) | 7±0 (4) |
| PNQ-318B | 33±0 (0) | 105±1 (1) | 212±1 (1) | | 689±8 (1) | 2±0 (5) |
| PNQ-302 | 1,154±7 (1) | 524±1 (0) | 953±11 (1) | | 2,597±18 (1) | N.D. |
| PNQ-332 | 3,856±29 (1) | 1,096±11 (1) | 1,067±18 (1) | | 2,654±14 (1) | 19±1 (5) |
| ATQ-270 | 89±2 (2) | 81±0 (0) | 76±2 (2) | | 69±0 (0) | 2±0 (5) |
| ATQ-284 | 31±2 (2) | 11±0 (1) | | 13±0 (2) | 35±0 (0) | N.D. |
| **%Recovery SAL-D_6_** | 84±0 (1) | 80±1 (1) | | 81±1 (2) | 84±0 (1) | 80±3 (4) |

N.D. = Not detected

**Table S4** Calibration parameters of the UPLC-MRM/MS method of the target analytes.

| Compounds | Regression equation | R^2^ | Linear range (µg/mL) |
| --- | --- | --- | --- |
| PNQ-288A | y = 0.5119x+0.0075 | 0.9998 | 0.014-5.76 |
| PNQ-288B | y = 0.5996x-0.0982 | 0.9972 | 0.014-2.88 |
| PNQ-318A | y = 0.8733x-0.0182 | 0.9999 | 0.016-3.18 |
| PNQ-318B | y = 0.7039x-0.0413 | 0.9998 | 0.016-3.18 |
| PNQ-302 | y = 0.7956x-0.2588 | 0.9958 | 0.015-6.04 |
| PNQ-332 | y = 0.8737x-0.2749 | 0.9956 | 0.017-6.64 |
| ATQ-270 | y = 0.5801x+0.0204 | 0.9979 | 0.014-0.54 |
| ATQ-284 | y = 2.2527x+0.0531 | 0.9989 | 0.014-0.57 |


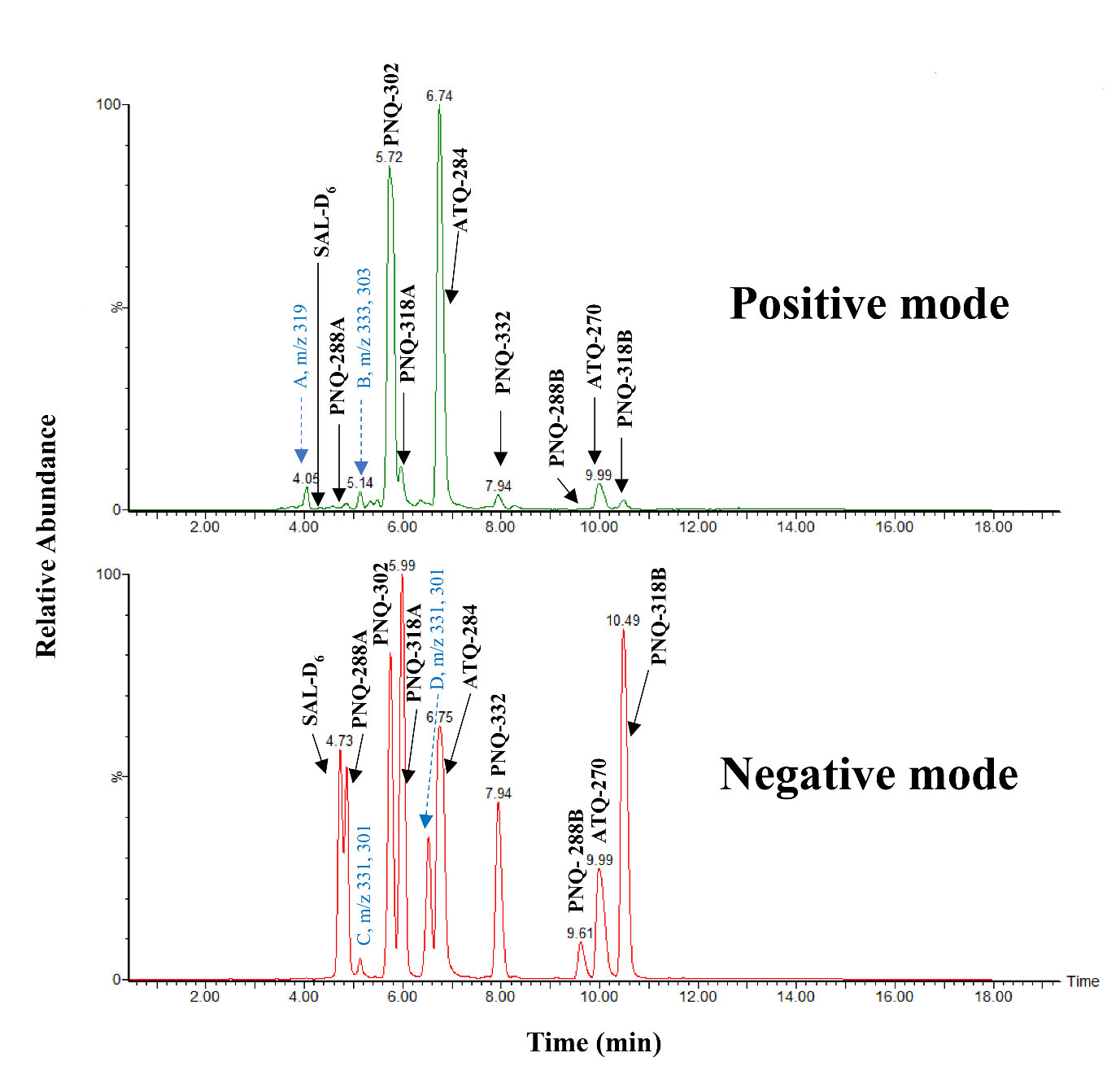


**Fig. S6** TIC comparison of signal responses in POS and NEG ESI modes obtained from a LC-MRM/MS analysis of unlabeled PNQ and ATQ standards in combination with the SAL-D_6_ (IS).

**
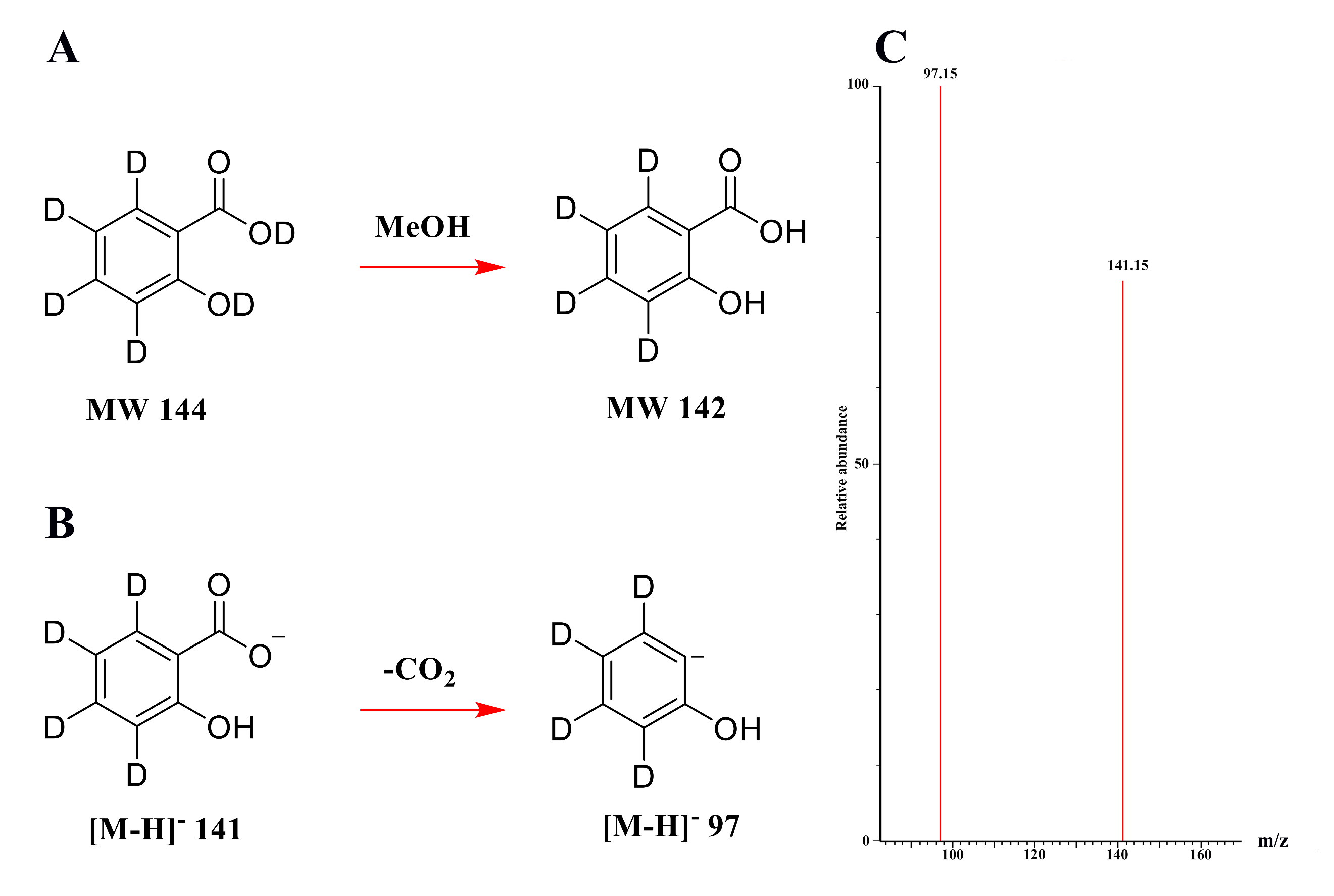
****Fig. S7** A. Reaction mechanism of SAL-D_6_ in MeOH. B. Fragmentation mechanism of SAL-D_6_ in NEG mode. C. MRM/MS spectrum of SAL-D_6_ in MRM mode (NEG).

**Table S5** Metabolite identification by RT, mass accuracy**.**

| **Compounds** | **Low-resolution MS (QQQ)** | | | **High-resolution (Q-TOF)** | | | |
| --- | --- | --- | --- | --- | --- | --- | --- |
|  | Standard  RT (min) | Heartwood sample RT (min) | %Accuracy  (RT) | Standard  RT (min) | Heartwood sample RT (min) | %Accuracy  (RT) | Mass accuracy (∆m, ppm) |
| **PNQ-288A** | 5.08 | 5.07 | 99.8 | 4.79 | 4.80 | 100.2 | 2.1 |
| **PNQ-288B** | 10.16 | 10.16 | 100.0 | 9.50 | 9.50 | 100.0 | 5.2 |
| **PNQ-318A** | 6.31 | 6.31 | 100.0 | 5.91 | 5.91 | 100.0 | 6.0 |
| **PNQ-318B** | 10.92 | 10.93 | 100.1 | 10.43 | 10.43 | 100.0 | 6.0 |
| **PNQ-302** | 6.03 | 6.04 | 100.2 | 5.68 | 5.67 | 99.8 | 1.0 |
| **PNQ-332** | 8.39 | 8.40 | 100.1 | 7.87 | 7.87 | 100.0 | -1.2 |
| **ATQ-270** | 10.52 | 10.53 | 100.1 | 9.94 | 9.94 | 100.0 | -3.0 |
| **ATQ-284** | 7.15 | 7.16 | 100.1 | 6.67 | 6.68 | 100.2 | -5.7 |


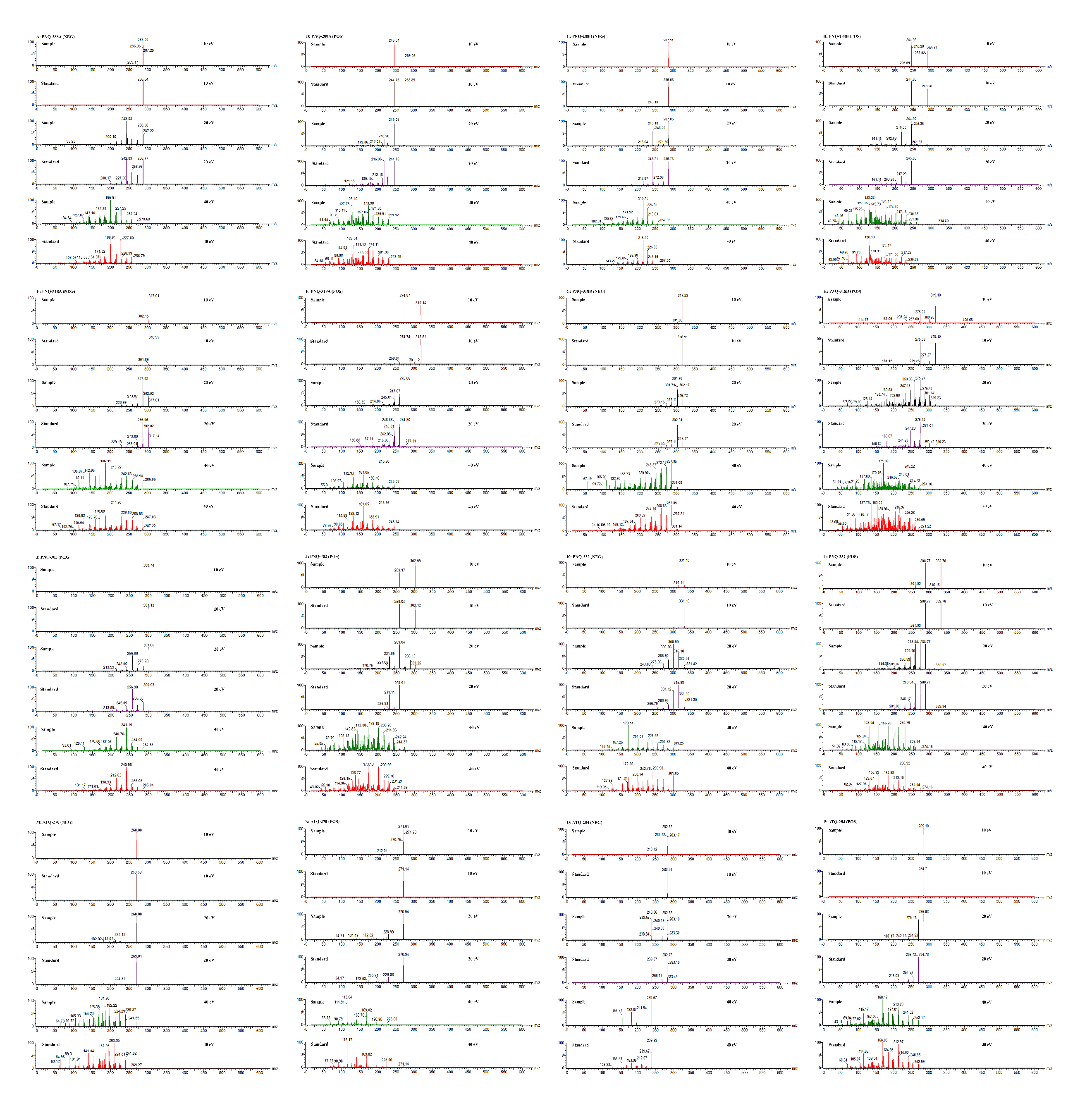


**A**

**B**

**C**

**D**

**E**

**F**

**G**

**H**

**I**

**J**

**K**

**L**

**M**

**N**

**O**

**P**

**Fig. S8** Comparison of the MS/MS spectra of all metabolites derived from PNQ and ATQ standards (20 µM) and a heartwood sample (10× dilution). The analysis was carried out using UPLC-MRM/MS in positive and negative ESI, with collision energies of 10, 20, and 40 eV; A: PNQ-288A (NEG), B: PNQ-288A (POS), C: PNQ-288B (NEG), D: PNQ-288B (POS), E: PNQ-318A (NEG), F: PNQ-318A (POS), G: PNQ-318B (NEG), H: PNQ-318B (POS), I: PNQ-302 (NEG), J: PNQ-302 (POS), K: PNQ-332 (NEG), L: PNQ-332 (POS), M: ATQ-270 (NEG), N: ATQ-270 (POS), O: ATQ-284 (NEG) and P: ATQ-284 (POS).

**Table S6** The % Recovery of SAL-D_6_ in spiking experiments.

| **Sample** | **%Recovery**  Calibration curve prepared in MeOH matrix (N=3) | **%Recovery**  Calibration curve prepared in *V. harmandiana* matrix (N=3) |
| --- | --- | --- |
| Heartwood | 0.6±0.1 | 84±4 |
| Wood | 2.0±0.1 | 116±4 |
| Bark | 3.0±1.0 | 110±4 |
| Root | 4.0±1.0 | 113±2 |
| Leaves | 4.0±0.3 | 128±1 |
